# Effects of resistance training and protein supplementation on bone turnover in young adult women

**DOI:** 10.1186/1743-7075-2-19

**Published:** 2005-08-17

**Authors:** Nicole M Mullins, Wayne E Sinning

**Affiliations:** 1 Department of Human Performance and Exercise Science, Youngstown State University, Youngstown, OH 44555, USA; 2 Exercise Science Laboratory, School of Exercise Leisure and Sport, Kent State University, Kent, OH 44242, USA

## Abstract

**Background:**

The strength of aging bone depends on the balance between the resorption and formation phases of the remodeling process. The purpose of this study was to examine the interaction of two factors with the potential to exert opposing influences on bone turnover, resistance exercise training and high dietary protein intake. It was hypothesized that resistance training by young, healthy, untrained women with protein intakes near recommended levels (0.8 g·kg^-1^·d^-1^) would promote bone formation and/or inhibit bone resorption, and that subsequent supplementation to provide 2.4 g protein·kg^-1^·d^-1 ^would reverse these effects.

**Methods:**

Bone formation was assessed with serum bone-specific alkaline phosphatase (BAP) and osteocalcin (OC), and bone resorption with urinary calcium and deoxypyridinoline (DPD). Biochemical, strength, anthropometric, dietary, and physical activity data were obtained from 24 healthy, untrained, eumenorrheic women (18–29y) at baseline, after eight weeks of resistance training (3 d·wk^-1^, ~1 hr·d^-1^; 3 sets, 6–10 repetitions, 13 exercises, 75–85% maximum voluntary contraction), and after 12 weeks of resistance training and 10 days of protein/placebo supplementation. Subjects were randomized (double-blind) to either a high protein (HP) or training control (TC) group and, during the final 10 days, consumed either enough purified whey protein to bring daily protein intake to 2.4 g·kg^-1^·d^-1^, or an equivalent dose of isoenergetic, carbohydrate placebo.

**Results:**

Strength, lean tissue mass, and DPD increased significantly in both groups over time, while percent body fat and BAP decreased (repeated measures ANOVA, p ≤ 0.05, Bonferroni correction). No significant changes were observed for serum OC or urinary calcium, and no significant group (TC, HP) × time (baseline, week 8, week 12) interactions emerged for any of the biochemical measures.

**Conclusion:**

(1) Twelve weeks of high-intensity resistance training did not appear to enhance bone formation or inhibit bone resorption in young adult women, as assessed by biochemical markers of bone metabolism. (2) Subsequent maintenance of a high protein intake for 10 days in these regularly-training, calcium-replete women also showed no effects on bone metabolism.

## Background

Maximizing genetic bone mass potential during youth, and minimizing bone loss throughout adulthood constitute osteoporosis prevention at its most fundamental level. To these ends, a great many factors in the pathogenesis of osteoporosis must be identified, assessed, and, as much as possible, offset. Certainly some prominent risk factors, such as age, sex, race, and family history are unalterable. Others, however, including diet, physical inactivity, menstrual status, medication use, low sunlight exposure, alcohol consumption, and cigarette smoking are amenable to change, and are therefore particularly important targets for osteoporosis prevention.

The purpose of this study was to examine the interaction of two lifestyle factors with the potential to exert opposing influences on bone metabolism: resistance training and high dietary protein intake. Some research indicates that resistance training has osteogenic, or bone-building effects, and some, that the metabolism of high protein loads has osteopenic, or bone-weakening effects. However, while these notions are commonly discussed as though they were well-established facts, the majority of research into both areas has come from cross-sectional or methodologically-limited experimental studies. Indeed, research has confirmed both the beneficial effects of weight-bearing activity [1] and the deleterious effects of a lack of it [2,3], but relatively few studies have specifically investigated the capacity of resistance training to *increase *bone mineral density (BMD) and mass. Among the few that have, findings have been inconsistent and clouded by a number of noteworthy limitations, including a lack of randomization [4-9] and the use of highly variable training intensities {≤ 10 repetitions and/or ≥ 70–85% one repetition maximum (1RM) [4,10-12]; ≥ 10 repetitions and/or ≤ 70% 1RM [5,6,9]}, subject populations (young, healthy subjects [4,5,8,11,12]; older individuals [6,7,9,10,13]; patients [14]), and study durations. Furthermore, because most have lasted fewer than 12 months, precision error is an important limitation in those that used bone densitometry. Since the annual rate of BMD change generally lies within the precision error of bone densitometry [15], it is difficult to accurately assess BMD gains or losses within fewer than several years.

Regarding dietary protein and bone strength, several cross-sectional studies have supported an inverse relationship [16-18], while others have not [19-21]. Experimental trials have also generated mixed findings [22,23], which are further complicated by the use of diverse subject populations, methods of assessing skeletal responses, experimental controls (e.g., calcium supplementation, menstrual status, etc.), and types, levels, and durations of protein supplementation. Still, despite these limitations, some authors have plainly labeled dietary protein a "negative risk factor" [[24], p. 336].

There are several theoretical mechanisms through which high protein intakes might stimulate bone demineralization, but the major possibility centers on the skeleton's constitution of an alkali reserve that can be called upon to assist in buffering the acidic catabolites of high-protein foods [25]. A complete discussion of this mechanism is beyond the scope of this article, but the body is known to mobilize calcium and phosphorus salts in the presence of high acid loads, and experimental increases in protein intake have been shown to stimulate bone resorption and calcium excretion [22,25-27]. Thus, the core concern is whether individuals who maintain chronically high protein intakes over-rely on skeletal buffers and incur preventable losses of bone mineral.

Investigation into possible interactive effects of resistance training and dietary protein on bone seems warranted because of the tendency of some people to regularly consume extraordinary amounts of protein. Traditionally, strength and bodybuilder athletes have been the most likely to follow high-protein diets; now many in the general population are following suit, due largely to the popularity of the various high-protein, weight-loss diets. Regardless of the rationale, it is known that some people habitually consume from three to more than five times [28-34] the adult recommended dietary allowance (RDA) for protein, despite clear evidence that such quantities constitute a nutritional overload [35,36]. An overload of dietary protein is generally indicated by increased rates of amino acid oxidation and urea nitrogen excretion. When protein is consumed in excess of the body's needs to synthesize new and replace degraded tissue proteins, it is not simply stored as such, but is broken down into its constituent amino acids. The surplus amino carbon fragments are then either oxidized for energy, or converted to carbohydrate or fat and stored, while the amino nitrogen fragments are excreted, mainly as urea in the urine.

Resistance exercise is known to stimulate muscle protein synthesis [37-40], which can thereby raise individual protein needs above the RDA of 0.8 grams per kilogram of body weight per day (g·kg^-1^·d^-1^) [41], to as much as much as 1.6 to 1.8 g·kg^-1^·d [42-45]. However, because protein synthesis eventually plateaus despite increasing dietary protein supply [43-45], no evidence supports a need for the extreme levels consumed by some athletes and dieters. It must be noted that these data assume adequate overall energy intake, as it has long been known that hypoenergetic diets increase protein requirements [46].

Thus, this double-blind, randomized trial was designed to compare the effects of a normal (i.e., near RDA) versus a high protein intake on bone metabolism in young adult women participating in regular, high-intensity resistance training. The high intake was set at 2.4 g·kg^-1^·d^-1 ^for several reasons: (1) several previous studies of protein intake and calcium balance have used values between 2.0 and 2.7 g·kg^-1^·d^-1 ^[22,23,26,47,48]; (2) several studies of strength and bodybuilder athletes have reported habitual protein intakes up to 4.3 g·kg^-1^·d^-1 ^[31-34]; (3) many people adhering to such plans as the Atkins Diet [28] and the Zone Diet [29] will consume well over 2.0 g·kg^-1^·d^-1^; and (4) it the first multiple of the RDA after 1.6 g·kg^-1^·d, which, as indicated, may be needed by some people [42-45]. A secondary objective was to examine bone turnover responses to the initiation of regular resistance training in untrained women with some remaining potential to add to skeletal mass (i.e., < 30 y) [49]. We hypothesized that the initiation of high-intensity resistance training in untrained, young adult women with protein intakes near recommended levels would stimulate osteogenic (i.e., increased bone formation) and/or anti-resorptive (i.e., reduced bone resorption) effects, and that a subsequent increase in protein intake would trigger osteopenic effects (i.e., increased bone resorption and/or reduced bone formation).

## Methods

### Experimental approach

The study was a two-group, 12-week, randomized trial. The first 10 1/2 weeks served as a control period, during which all of the subjects followed the same relative resistance training program, maintained their usual diets, and consumed a daily calcium supplement. Biochemical, strength, anthropometric, dietary, and physical activity data were collected at three time points: baseline (prior to both resistance training and protein/placebo supplementation), week 8 (after initial adaptation to resistance training, but prior to supplementation), and week 12 (after continued resistance training and 10 days of supplementation). This timeline was planned largely to control for menses-related variability in the biochemical measures and to fit within a semester at the university. Considering that both protein utilization [50] and bone biomarker concentrations [51] may vary throughout the menstrual cycle, blood and urine sampling at each time point was scheduled during the early follicular phase (days 1–8). This essentially left data collection opportunities for each woman at approximately weeks 4, 8, and 12. Week 4 was not used, to allow an initial period of adaptation to training to pass before applying the experimental treatment (i.e., protein supplement/placebo). While resistance training could initiate detectable changes in the biomarkers before week 8, the aim of the study was to investigate effects of mechanical loading and protein supplementation on the markers, not effects of the novel exercise. Since previous research has shown that the early stages of resistance training may increase dietary protein needs [34,42,45], the consumption of supplemental protein soon after the initiation of an exercise program might not induce as great an acid effect. Week 16 was not considered, because semester-end schedule changes and relocations were likely to prevent the continued participation of many subjects.

### Subjects

Thirty healthy, eumenorrheic women between 18 and 30 years of age were recruited from a general university population to form a training control (TC) group and a high protein (HP) group. Exclusion criteria included having fewer than 10 normal menses during the previous year, engaging in resistance training within two years, using any drugs or medications known to affect bone metabolism within six months, having any known metabolic bone disease or chronic physical condition that would limit participation in an exercise program, and regularly smoking more than 10 cigarettes or drinking more than two alcoholic beverages per day. These criteria were established on the basis of research documenting their potential to affect bone [52] and their inclusion in related studies [11,12]. Race was not restricted because a repeated measures design was used, and because there is no evidence suggesting that women with different racial backgrounds would respond differently to training. The study was approved by the Kent State University Institutional Review Board, and informed consent was obtained from all subjects prior to data collection.

### Data collection protocol

Of the more than 150 women who responded to the initial request for study volunteers, the first 30 to satisfy the eligibility criteria and agree to all procedures and responsibilities were accepted. Each subject reported to the Exercise Science Laboratory four times. During the initial visit, each completed the health history and physical activity questionnaires and underwent an anthropometric assessment. Preconditions for the anthropometry were explained via electronic mail or telephone prior to the initial visit, including appropriate attire and avoidance of any substances or activities that could significantly alter hydration status (specifically, heavy exercise for 12 hours, caffeine for 24 hours, alcohol for 48 hours). Each subject then received detailed instructions on keeping accurate seven-day diet records and collecting 24-hour urine specimens, and scheduled a second laboratory visit during her next anticipated follicular phase (visits were rescheduled if menses started earlier or later than anticipated). Finally, to exclude the potential effects of calcium deficiency, each subject was given a supply of calcium supplements (Tums Calcium for Life™-Bone Health, SmithKline Beecham, Pittsburgh, PA) to begin consuming for the duration of the study. Each was instructed to carry the calcium tablets in her purse or backpack, and was regularly reminded to consume one 500-mg tablet, twice per day.

For each subsequent laboratory visit, subjects reported between 6:00 and 9:00 a.m. in the overnight fasted state to control for circadian, dietary, and exercise influences on the biochemical measures. Of these, circadian variability appears to have the greatest impact, with most serum and urinary biomarker levels being unaffected by diet and only slightly affected by prior exercise [53]. Diet records and urine specimens were collected and a venous blood sample drawn for the determination of serum OC, BAP, E_2_, and P_4_. The serum was immediately separated from the blood cells, transferred into separate Cryovials^® ^(Nalgene, Rochester, NY) for each biomarker, and stored at -120°C. Total urine volume was measured and several aliquots frozen at -120°C (DPD) and -20°C (calcium, creatinine, pH, urea nitrogen).

### Questionnaires

Questionnaires were administered to assess general health, menstrual, and physical activity histories and to monitor ongoing health status and physical activity levels. The health questionnaire was a standard form of the Exercise Science Laboratory, supplemented with questions on menstrual history and eating behaviors. The physical activity questionnaire (PAQ) was based on the Seven-Day Activity Recall of Sallis et al. [54], and used to determine whether the subjects' participation in resistance training affected their involvement in other types of physical activity.

### Anthropometry

Skinfold thicknesses (mm) were measured with Holtain^® ^calipers (Holtain Ltd., Wales, UK) at the subscapular, triceps, chest, midaxillary, anterior suprailiac, abdominal, and mid-thigh sites, and used to estimate body composition. Percent body fat (%BF), fat mass (FM), and fat-free mass (FFM) were derived using the Jackson et al. [55] seven-site skinfold equation for adult women and the body density formula of Brozek et al. [56]. Girths were obtained with a fiberglass measuring tape at the neck, shoulders, chest, waist, umbilicus, hips, gluteal thigh, mid-thigh, calf, mid-arm, and forearm to evaluate the effects of resistance training on muscle mass. All values were obtained in triplicate according to Lohman et al. [57], by one experienced anthropometrist (N. Mullins). Median values were used as data.

### Diet analyses

All subjects completed seven-day diet records prior to laboratory visits 2–4. Baseline and week 8 records were used to assess habitual dietary intake, consistency, and any between-groups differences. Week 12 records were used to confirm the subjects' continued maintenance of their usual diets. Estimates of average daily intake were computed for total kilocalories (kcals), percent kcals (%kcals) from protein, carbohydrate, fat, and alcohol, grams of protein per kilogram of body weight (g·kg^-1^), and calcium (mg), phosphorus (mg), vitamin D (μg), sodium (mg), magnesium (mg), caffeine (mg), and fiber (g) using diet analysis software (Diet Analysis Plus^©^, Version 4.0, ESHA Research, Salem, OR).

### Biochemical measures

Serum osteocalcin (OC) and serum bone-specific alkaline phosphatase (BAP) were used as indicators of bone formation, and urinary deoxypyridinoline (DPD) and urinary calcium as indicators of bone resorption. Detailed discussions of the individual markers are available in several reviews [53,58-60]. The bone markers and urinary creatinine (correction for urinary DPD) were measured at all three time points, and serum estradiol (E_2_) and progesterone (P_4_) at baseline to confirm menstrual status.

Serum OC (Metra™ Osteocalcin), serum BAP (Metra™ BAP), and urinary DPD (Metra™ DPD) were measured with enzyme-linked immunosorbant assay kits (Quidel Corporation, Santa Clara, CA), and serum E_2 _(Coat-A-Count^® ^Estradiol) and P_4 _(Coat-A-Count^® ^Progesterone) via radioimmunassay (Diagnostics Products Corporation, Los Angeles, CA). Urinary calcium and creatinine were measured using automated procedures at Suburban Medical Laboratory (Cuyahoga Falls, OH). To minimize interassay variation, all serum and urine samples remained in frozen storage until all data were collected and assays could be run simultaneously. Duplicate values for all biochemical measures were obtained and mean values used as data.

### Strength testing and training

Between the first and second laboratory visits, each subject was familiarized with the resistance training facility (in the same building as the laboratory) and given individual instruction on training safety and techniques. Each completed two familiarization workouts under one-on-one supervision, after which baseline strength testing was scheduled (at least 48 hours later). Strength was evaluated using 1RM procedures for select exercises (Table 1), and also using isometric dynamometry, to provide strength scores independent of the training program.

**Table 1:** Exercises used for resistance training and strength testing

**MUSCLE GROUP**	**EXERCISES**	**MUSCLE GROUP**	**EXERCISES**
**Back**	Lat pull-down^a^ Bent-over dumbbell row Seated row Underhand pull-down	**Biceps**	Seated dumbbell curl^a^ Concentration curl Preacher curl Hammer curl
**Quadriceps**	Leg extension^a^ Seated leg press Dumbbell squats Dumbbell lunges	**Triceps**	Triceps pressdown^a^ Triceps kickback Overhead triceps extension Triceps dips
**Chest**	Chest press^a^ Dumbbell flyes Dumbbell bench press Dumbbell pullover	**Calves**	Standing calf raise^a^ Seated plantar flexion Single-leg standing calf raise
**Hamstrings**	Lying hamstring curl^a^ Deadlifts	**Upper Body**	Push-ups^b^ Assisted chin-ups Assisted dips
**Shoulders**	Overhead press^a^ Lateral raises Front raises Shrugs	**Lower Back**	Machine back extension Roman chair
**Hip**	Standing hip abduction^a^ Seated hip abduction Standing hip extension	**Abdominals**	Basic crunches Reverse crunches Elbow crunches Machine crunches Hanging knee-ups Oblique knee-ups
**Hip**	Standing hip adduction^a^ Seated hip adduction Standing hip flexion		

All underlined exercises were used during weeks 1, 2, 5, 8, 11, 12, with alternative exercises used during the other weeks to provide variety and enhance adherence.

^a^Exercises used in 1-RM testing

^b ^Tested for maximum number of repetitions

Peak isometric flexion and extension force of the right arm and leg were determined using an electronic dynamometer, designed and built by Karpovich and Karpovich [61] and interfaced with a Biopak^® ^computer system and AcqKnowledge^® ^software (Biopak^® ^Systems Inc, Santa Barbara, CA). Voltage readings from the dynamometer were converted to force units (kg) by the software, and peak force values easily identified from force curves. Subject instruction, encouragement and positioning, including posture, joint angle and limb stabilization, were closely controlled. The subjects' limbs were positioned at the joint angles shown to permit maximum force production: 115° for elbow flexion, 40° for elbow extension, 165° for knee flexion, and 115° for leg extension [62]. Three trials for each movement were performed, and the median values used as data.

Once baseline strength and all other baseline measures were obtained (lab visit 2), each subject began her 12-week program of high-intensity resistance training targeting the major muscle groups (3 d·wk^-1^, ~1 hr·d^-1^, 3 sets of 6–10 repetitions for 13 different exercises at 75–85% of 1-RM values; Table 1). All training sessions were fully supervised (subject:instructor ratios between 1:1 and 4:1), and subjects' training loads continually adjusted to maintain proper intensity levels.

### Protein/placebo supplementation

During week 10, all subjects were randomized (double-blind) to either the TC or HP group. HP subjects were provided with a 10-day supply of purified whey protein (Extreme Pure Pro™, American Body Building Products^®^, Waterboro, SC), and TC subjects with a 10-day supply of carbohydrate placebo (Maltrin^® ^M100 Maltodextrin, Grain Processing Corporation, Muscatine, IA). Treatment dosages were measured, packaged, and distributed by a single, third party technician. Daily rations were prepared to bring each HP subject's average protein intake to 2.4 g·kg^-1^·d^-1^, or to supply each TC subject with an equivalent dose of placebo. They were provided in three to five small containers, with the recommendation to consume one drink with each daily meal, and any additional supplement *ad libitum*. Since the protein supplement was a fruit punch flavored concentrate and the placebo an unflavored powder, the placebo was mixed with enough fruit drink powder (carbohydrate only) to make it similar in taste, appearance, and energy content to the protein supplement. The subjects had only to add water to prepare either substance. Each subject consumed the assigned daily ration during the final 10 days of her training program, and reported for week 12 data collection immediately afterwards (day 11).

### Statistical Analyses

Descriptive statistics were computed for all data and select health questionnaire responses were evaluated in terms of frequency and percent frequency. All data were checked for normality and nonparametric tests were used where appropriate. Data were analyzed for the main effects of group and time and the interaction of group by time with a two (group: HP, TC) × three (test period: baseline, week 8, week 12) repeated-measures analysis of variance (ANOVA). Group differences in diet composition and exercise adherence were examined using Mann-Whitney tests, and Wilcoxon Signed Rank tests were used to test within-groups consistency in dietary intake over time. Significance was set at the *p *≤ 0.05 level for all comparisons, and the Bonferroni correction used where multiple comparisons were made. Data were analyzed using SPSS for Windows, Version 9.0 (SPSS Inc., Chicago, IL, USA).

## Results

### Subject Characteristics and Exercise Adherence

Six volunteers dropped out within the first week of the study (three due to schedule conflicts and three due to unrelated health concerns), leaving a total of 24 subjects who completed the study (12 TC, 12 HP). Baseline subject characteristics for the TC and HP groups are shown in Table 2. There were no initial, between-groups differences in age, height, weight, %BF, FFM, FM, or serum E_2 _or P_4_. All subjects had baseline serum E_2 _and P_4 _levels within normal follicular phase ranges (i.e., E_2 _10–200 pg·ml^-1^; P_4 _0.15–1.4 ng·ml^-1^; Diagnostics Products Corporation, Los Angeles, CA) and maintained menstrual function throughout the study. The same number of women in each group used oral contraceptives (n = 7). Attendance at the resistance training sessions was excellent and similar for both groups (TC 92%, HP 94%, *p *= 0.86). On average, subjects in both groups missed only three of approximately 36 total training sessions. The total possible number of training sessions slightly exceeded 36 (36.6), as it was important to maintain the trained state while scheduling final data collection around slight menstrual cycle delays.

**Table 2:** Baseline subject characteristics (mean ± SE) of the training control (TC) and high-protein (HP) groups

**VARIABLE**	**TC (n = 12)**	**HP (n = 12)**
**Age (yr)**	22.7 ± 1.07	22.8 ± 0.85
**Height (cm)**	164.9 ± 1.84	167.1 ± 1.75
**Weight (kg)**	64.8 ± 2.31	64.2 ± 2.48
**Body fat (%)**	27.9 ± 1.70	26.0 ± 1.96
**FFM (kg)**	46.4 ± 1.00	47.1 ± 1.25
**FM (kg)**	18.4 ± 1.68	17.1 ± 1.81

* Significant (*p *≤ 0.05) difference, independent samples t-tests

### Dietary Intake

Both groups maintained similar and consistent diets over time, with no significant differences between groups at any time point, and no significant changes over time in any of the assessed dietary variables. Baseline and week 8 dietary data were pooled to provide the basis for calculating supplement dosages (Table 3).

**Table 3:** Mean^† ^daily nutrient intake (mean ± SE) for the training control (TC) and high-protein (HP) groups

**VARIABLE**	**TC (n = 12)**	**HP (n = 12)**
**Energy (kcals)**	1632 ± 141	1792 ± 103
**Protein (g·kg**^-1^**)**	0.96 ± 0.05	0.91 ± 0.07
**Protein (% kcals)**	15.4 ± 1.0	12.9 ± 0.5
**Carbohydrate (% kcals)**	56.2 ± 2.2	55.9 ± 1.6
**Fat (% kcals)**	27.9 ± 1.5	27.2 ± 1.4
**Alcohol (% kcals)**	1.1 ± 0.5	3.2 ± 1.0
**Calcium (mg)**	685 ± 84	682 ± 64
**Phosphorus (mg)**	873 ± 75	876 ± 56
**Vitamin D (μg)**	3.37 ± 0.53	2.50 ± 0.38
**Magnesium (mg)**	182 ± 17	209 ± 13
**Fiber (g)**	12.4 ± 4.5	15.1 ± 5.9
**Sodium (mg)**	2428 ± 211	2781 ± 158
**Caffeine (mg)**	56 ± 16	46 ± 16

* Significant (*p *≤ 0.05) difference, Mann-Whitney tests

^† ^14-day means

### Physical Activity Questionnaire Data

No significant group by time interactions emerged from the PAQ data (not shown). Logically, there was a significant main effect for time (*p *≤ 0.001) in the number of weekly hours spent performing strength exercise, but time spent engaged in all other types and levels of physical activity did not change significantly (moderate, hard or very hard physical activity, flexibility exercise, sleeping). There was a significant group effect in moderate physical activity (*p *= 0.02), such that the TC group reportedly engaged in more moderate-intensity physical activity than the HP group, but at all time points and not as a result of the resistance training or dietary protein interventions.

### Strength and Body Composition

**Figure 1:**
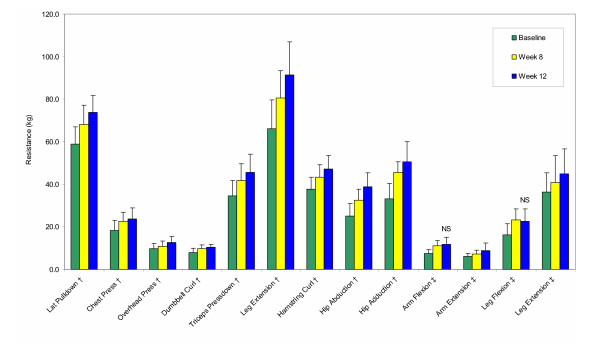
Strength changes over time (mean ± SE) for the training control (TC) and high-protein (HP) groups combined (n = 24). Absolute strength was similar between TC and HP at all time points, and strength increases were significant (p ≤ 0.05) between all time points for all measures, except for those of isometric arm and leg flexion, which were not significantly different between weeks 8 and 12 (NS). ^† ^One-repetition (1-RM) maximum (kg) ^‡ ^Isometric dynamometry (kg)

**Figure 2:**
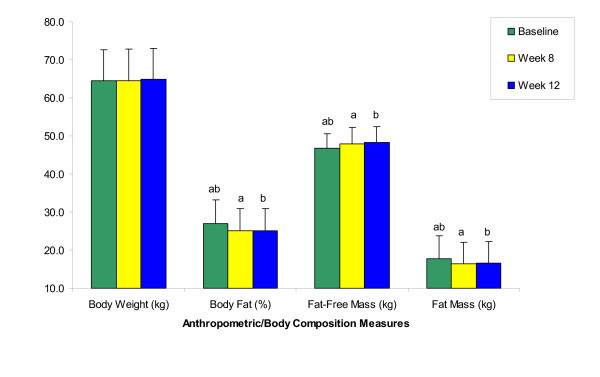
Anthropometric and body composition measures (mean ± SE) for the training control (TC) and high-protein (HP) groups combined (n = 24). All measures were similar between TC and HP at all time points, with both groups showing similar, significant (*p *≤ 0.05) reductions in percent body fat and gains in lean tissue mass over time. ^ab ^Like letters are significantly different.

Large increases in voluntary strength (Figure 1) and changes in body composition (Figure 2) confirm the effectiveness of the training program. Since all strength and body composition measures were initially similar between groups and progressed similarly throughout the study, these data are presented as total sample means. Percent increases in strength over the course of the 12-week program ranged from 26–143% for upper body measures, and from 25–83% for lower body measures. Strength improvements were significant between all time points for all strength measures, except for those of isometric arm and leg flexion, which did not increase significantly between weeks 8 and 12. With regard to body composition, both groups showed similar, significant reductions in percent body fat (TC -6.5%, HP -7.3%, *p *≤ 0.001) and gains in lean tissue mass (TC 2.7%, HP 3.9%, *p *≤ 0.001).

### Biochemical Measures

**Figure 3:**
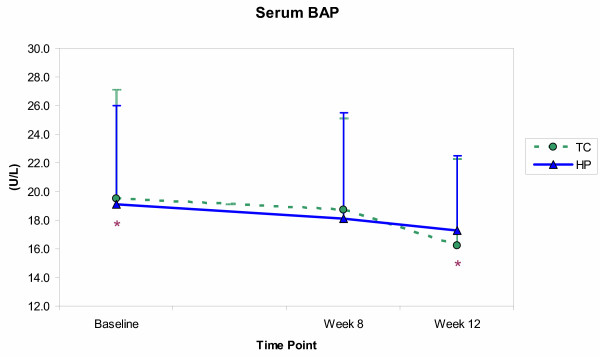
Serum bone-specific alkaline phosphatase (BAP) concentrations (mean ± SE) for the training control (TC) and high-protein (HP) groups from baseline through week 12. For both groups, BAP levels were significantly (*p *≤ 0.05) lower at week 12 than at baseline. * Time point values are significantly different.

**Figure 4:**
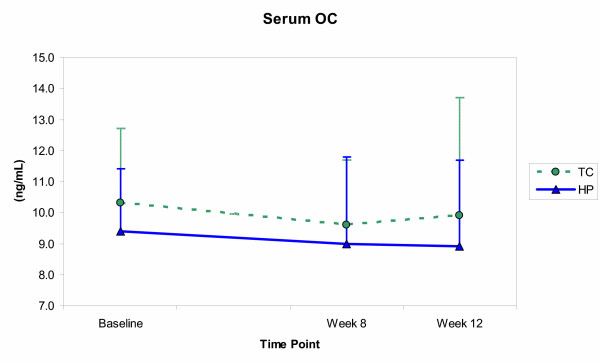
Serum osteocalcin (OC) concentrations (mean ± SE) for the training control (TC) and high-protein (HP) groups from baseline through week 12. Differences were not significant (*p *≤ 0.05) for group, for time, or for group × time effects.

**Figure 5:**
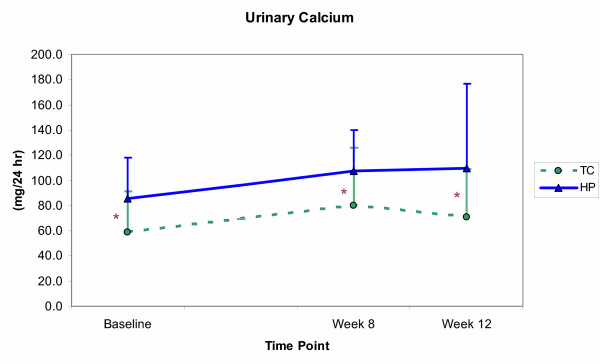
Urinary calcium concentrations (mean ± SE) for the training control (TC) and high-protein (HP) groups from baseline through week 12. Calcium levels were significantly (*p *≤ 0.05) greater in TC than HP at all time points. * Group values are significantly different.

**Figure 6:**
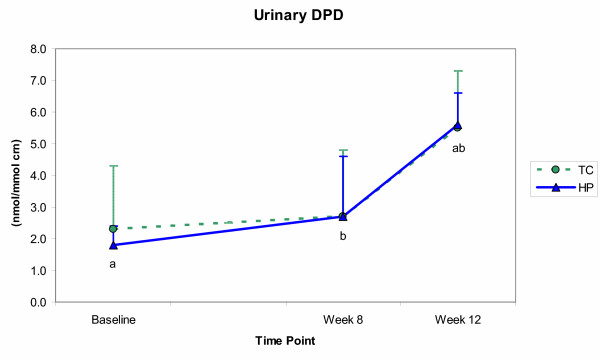
Urinary deoxypyridinoline (DPD) concentrations (mean ± SE) for the training control (TC) and high-protein (HP) groups from baseline through week 12. For both groups, DPD levels were significantly (*p *≤ 0.05) higher at week 12 than at both baseline and week 8. ^ab ^Like letters are significantly different.

The effects of resistance training and protein intake on the biochemical measures are shown in Figures 3, 4, 5, 6. Significant time effects were observed for serum BAP and urinary DPD in both groups, such that BAP was significantly lower at week 12 than at baseline (*p *≤ 0.001), while DPD was significantly higher at week 12 than at both baseline and week 8 (*p *≤ 0.001). A significant group effect emerged for urinary calcium, with the HP group showing greater calcium excretion than the TC group (*p *= 0.02), but at all time points and not as a result of training or supplementation. Serum OC showed neither time, nor group effects, and no significant group by time interactions emerged for any of the biochemical measures.

## Discussion

The strength of aging bone depends on the balance between the resorption and formation phases of the remodeling process. To our knowledge, this is the first study to examine the interaction of two lifestyle factors with the potential to exert opposing influences on bone turnover, resistance exercise training and high dietary protein intake. It was hypothesized that high-intensity resistance training would increase bone formation and reduce bone resorption activity, as indicated by biochemical markers of bone metabolism, and that subsequent consumption of a high protein intake would reverse these effects. The loading intensity was at least as great as that in previous studies which reported significant osteogenic responses to resistance exercise [10-12], and the protein intake was at least as great as that in previous studies which reported significant protein-induced calciuric effects [47,48,63].

The results, however, conflicted with several previous findings. Progressive resistance training did not prompt an increase in formation marker concentrations, with serum BAP declining and serum OC showing no change in both groups over time. In contrast, Fujimura et al. [4] reported significantly elevated BAP and OC levels in eight healthy, young men (23–31y) one month after initiating resistance training – levels that remained elevated through four months of training and that were not seen in seven age-matched, non-exercising controls. Menkes et al. [6] reported significant increases in BAP and OC after 12 and 16 weeks of resistance training, respectively, in 11 healthy, untrained, older men (59 ± 2y), but not in seven non-training controls. Rockwell et al. [8] and Nelson et al. [10] reported significantly elevated OC levels after initiating resistance training in eumenorrheic, premenopausal (mean ± SE, 36.2 ± 1.3 yr) and postmenopausal (50–70 y) women, respectively. While the exercise protocols and subject samples varied, these studies all supported the possibility that resistance training might stimulate osteogenic effects, detectable through changes in bone formation marker concentrations.

Though explanations for the discrepant findings are not clear, for several reasons, we are confident that neither exercise adherence, nor training intensity was a confounding factor. First, the subjects' high overall attendance at the training sessions (93%) and their significant strength and body composition changes throughout the study (Figures 1 and 2) support the effectiveness of the exercise program. Second, the training program was consistent with recently published recommendations by the American College of Sports Medicine for preserving bone health throughout adulthood [64]. Third, all workouts were fully supervised, resistances carefully monitored and adjusted, and personalized encouragement given to help each subject consistently train at target levels. If resistance training can stimulate osteogenic responses in young adult women, the present program should have provided an adequate stimulus to do so.

The few studies that have evaluated the effects of resistance training on both biomarker concentrations and BMD were particularly important to forming the hypothesis that high-intensity resistance training would stimulate osteogenic shifts in the bone metabolism of previously untrained subjects. An overview of the majority of these studies is presented in Table 4[4-11,13,65]. However, while importantly contributing to the literature, these studies are complicated by several limitations, including nonrandomized designs, short study durations, low-intensity training protocols, small and varied subject samples (age, sex, previous training), inadequate controls over important hormonal and nutritional factors (estrogen, calcium), and the use of different bone assessment techniques. In fact, six of the 10 studies cited allowed subjects to self-select membership to exercise or control groups – all six of which reported either BMD or biomarker changes that could be interpreted as beneficial effects of resistance training. Since there may be inherent differences that motivate some individuals to choose exercise over control participation, these results must be viewed cautiously.

**Table 4:** Longitudinal studies of resistance training (RT) effects on bone mineral density (BMD^1^) and biochemical markers of bone metabolism


**Bemben et al., 2000**	**Subjects: **25 postmenopausal women (17 RT^1^, 8 C), 41–60 y **Eligibility criteria: **No RT previous 6 months; no current or recent HRT **Design: **Randomized (subjects matched for lumbar BMD, then randomly assigned to groups) **Training duration / frequency: **6 months / 3 d·wk^-1 ^(~45 min·d^-1^) **Training intensity: **3 sets, 8–16 reps, 40–80% 1-RM, 12 exercises **Calcium supplementation: **600 mg·d^-1^+125 IU vit. D only for subjects with daily Ca^2+ ^<1500 mg **Training supervised: **Yes Results-Strength: * Strength ↑ in RT, but not in C **Results-BMD: **Total body, lumbar, & femoral BMD changes – NS **Results-Biomarkers: **OC & CTX changes – NS

**Fujimura et al., 1997**	**Subjects: **15 adult men (8 RT, 7 C), 23–31 y **Eligibility criteria: **No RT previous 2 y **Design: **Not randomized **Training duration / frequency: **4 months / 4 d·wk^-1 ^(~45 min·d^-1^) **Training intensity: **1 set, 10 reps, 60% 1-RM, & 2 sets, 10 reps, 80% 1-RM, 7–8 exercises **Calcium supplementation: **600 mg·d^-1^ **Training supervised: **Not indicated Results-Strength: * Strength ↑ in RT, but not in C **Results-BMD: **Total body, lumbar, femoral, radial BMD changes – NS Results-Biomarkers: * OC & BAP ↑ at 1, 2, 3, & 4 months in RT, but not in C * PICP ↓ at 2 & 4 months in C, but not in RT (not measured at 3 months in C) DPD changes – NS

**Gleeson et al., 1990**	**Subjects: **72 eumenorrheic women (34 RT, 38 C), 23–46 y **Eligibility criteria: **No previous, regular RT; oral contraceptive users eligible **Design: **Not randomized **Training duration / frequency: **12 months / 3 d·wk^-1 ^(~30 min·d^-1^) **Training intensity: **2 sets, 20 reps, 60% 1-RM, 8 exercises **Calcium supplementation: **500 mg·d^-1^ **Training supervised: **Not indicated Results-Strength: * Strength ↑ in RT, but not in C Results-BMD: * Percent lumbar BMD change (0.8% ↑ in RT vs. 0.5% ↓ in C) Absolute lumbar (DPA) & calcaneal (SPA) BMD changes – NS **Results-Biomarkers: **OC changes – NS

**Lohman et al., 1995**	**Subjects: **56 eumenorrheic women (22 RT, 34 C), 28–39 y **Eligibility criteria: **No RT previous 2 y; oral contraceptives users not eligible **Design: **Randomized **Training duration / frequency: **18 months / 3 d·wk^-1 ^(~1 hr·d^-1^) **Training intensity: **3 sets, 8–12 reps, 70–80% 1-RM, 12 exercises **Calcium supplementation: **500 mg·d^-1^ **Training supervised: **Yes Results-Strength: * Strength ↑ in RT, but not in C Results-BMD: * Lumbar BMD ↑ at 5 (2.0%) & 12 (1.6%) months in RT, but not in C * Trochanteric BMD ↑ at 5 (0.8%), 12 (2.4%), & 18 (1.5%) months in RT, but not in C Total body & radial BMD (SPA) – NS Results-Biomarkers: * OC ↑ in RT at 5, 12, & 18 months, but not in C

**Menkes et al., 1993**	**Subjects: **18 men (11 RT, 7 C), 50–70 y **Eligibility criteria: **No RT previous 2 y **Design: **Not randomized **Training duration / frequency: **4 months / 3 d·wk^-1^ **Training intensity: **1 set, 15 reps, 8 upper body exercises, & 2 sets, 15 reps, 4 lower body exercises **Calcium supplementation: **No: subjects instructed to follow diet containing 1000 mg·d^-1 ^calcium **Training supervised: **Yes Results-Strength: * Strength ↑ in RT, but not in C Results-BMD: * Lumbar (2.0%) & femoral (3.8%) BMD ↑ in RT, but not in C Total body BMD changes – NS Results-Biomarkers: * OC ↑ at 3 & 4 months, & BAP ↑ at 4 months in RT, but not in C TRAP changes – NS

**Nelson et al., 1994**	**Subjects: **39 postmenopausal women (20 RT, 19 C), 50–70 y **Eligibility criteria: **No current regular exercise; no HRT previous 12 months **Design: **Randomized **Training duration / frequency: **12 months / 2 d·wk^-1 ^(~45 min·d^-1^) **Training intensity: **3 sets, 8 reps, 80% 1-RM, 5 exercises **Calcium supplementation: **No: subjects consuming < 800 mg·d^-1 ^counseled to ↑ intake **Training supervised: **Yes Results-Strength: * Strength ↑ in RT, but not in C Results-BMD: * Lumbar (1.0%) & femoral (0.9%) BMD ↑ in RT, but ↓ in C (lumb. -1.8, fem. -2.5%) Total body BMD changes – NS Results-Biomarkers: * OC ↑ in RT, but ↓ in C

**Pruitt et al., 1992**	**Subjects: **26 postmenopausal women (17 RT, 9 C), RT 53.6 ± 1.0 y, C 55.6 ± 0.9 y **Eligibility criteria: **No RT previous 6 months; no previous HRT **Design: **Not randomized (early respondents placed in RT group) **Training duration / frequency: **9 months / 3 d·wk^-1 ^(~40 min·d^-1^) **Training intensity: **1 set, 10–15 reps, 11 exercises **Calcium supplementation: **Not indicated **Training supervised: **Not indicated Results-Strength: * Strength ↑ in RT, but not in C Results-BMD: * Lumbar BMD (DPA) ↑ in RT (1.6%), but ↓ in C (-3.6%) Femoral (DPA) & forearm (SPA) BMD changes – NS Results-Biomarkers: * Baseline OC greater in RT than in C OC, BAP, & HYP changes – NS

**Pruitt et al., 1995**	**Subjects: **26 postmenopausal women (15 RT^2^, 11 C), 65–79 y **Eligibility criteria: **No previous RT; no previous HRT or HRT ≥ 1 y **Design: **Randomized **Training duration / frequency: **12 months / 3 d·wk^-1 ^(~50–55 min·d^-1^) **Training intensity: **3 sets, 7–14 reps, 40–80% 1-RM, 10 exercises **Calcium supplementation: **500 mg·d^-1^+200 IU vitamin D **Training supervised: **Yes Results-Strength: * Strength ↑ in RT, but not in C **Results-BMD: **Lumbar & hip BMD changes – NS **Results-Biomarkers: **OC changes – NS

**Rockwell et al., 1990**	**Subjects: **17 eumenorrheic women (10 RT, 7 C), RT 36.2 ± 1.3 y, C 40.4 ± 1.6y **Eligibility criteria: **No previous RT; oral contraceptive users not eligible **Design: **Not randomized **Training duration / frequency: **9 months / 2 d·wk^-1 ^(~45 min·d^-1^) **Training intensity: **2 sets, 12 reps, 70% 1-RM, 8 exercises **Calcium supplementation: **500 mg·d^-1 ^+200 IU vitamin D **Training supervised: **Not indicated Results-Strength: * Strength ↑ in RT, but not in C Results-BMD: * Lumbar BMD ↓ in RT at 4.5 (-2.9%) & 9 months (-4.0%); no change in C Femoral BMD changes – NS Results-Biomarkers: * Baseline OC greater in RT than in C * OC ↑ in RT & in C at 4.5 & 9 months

**Ryan et al., 1994**	**Subjects: **37 men (21 RT, 16 C), 51–71 y **Eligibility criteria: **No RT previous 6 months **Design: **Not randomized **Training duration / frequency: **4 months / 3 d·wk^-1^ **Training intensity: **1 set, 15 reps using variable resistance machines, 14 exercises **Calcium supplementation: **Not indicated **Training supervised: **Not indicated Results-Strength: * Strength ↑ in RT, but not in C Results-BMD: * Femoral BMD ↑ in RT (2.8%), but not in C Total body & lumbar BMD (DXA) changes – NS Results-Biomarkers: * TRAP ↑ in RT & in C (no difference between groups) Changes in OC & BAP – NS

BMD^1 ^= all BMD measurements made using dual-energy x-ray absorptiometry (DXA) unless otherwise specified (DPA = dual-photon absorptiometry; SPA = single-photon absorptiometry)

RT = resistance training

C = control

RT^1 ^= two training groups: 10 high-load RT & 7 high-repetition RT

HRT = hormone replacement therapy

* = statistically significant

NS = non-significant

OC = serum osteocalcin

CTX = urinary C-telopeptide

BAP = serum bone-specific alkaline phosphatase

DPD = urinary deoxypyridinoline

PICP = plasma carboxy-terminal pro-collagen 1 extension peptide

TRAP = serum tartrate resistant acid phosphatase

HYP = urinary hydroxyproline

RT^2 ^= two training groups: 8 high-intensity RT & 7 low-intensity RT

Particularly notable among the first studies to use both biomarker technology and densitometry to examine resistance training effects is that of Lohman et al. [11], due to its inclusion of important controls for hormonal status, training intensity, and calcium intake. Lohman's group reported significantly elevated serum OC in 22 young adult women (28–39 y) after five, 12, and 18 months of high-intensity resistance training, but not in 34 age-matched, non-training controls. Since the present study used both a similar subject sample and training program (Lohman et al.: 1 h·d^-1^, 3 d·wk^-1^, 3 sets, 8–12 repetitions, 12 exercises, 70–80% 1RM; present study: ~1 h·d^-1^, 3 d·wk^-1^, 3 sets, 6–10 repetitions, 13 exercises, 75–85% 1RM), similar results were anticipated. The present study was shorter, but did produce significant strength and body composition changes and was markedly longer than other studies in which biomarker responses to experimental manipulations have been observed [22,23,66].

Even fewer studies have specifically addressed the possibility that resistance training may benefit bone by reducing bone resorption. Fujimura et al. [4], cited earlier, reported not only significant elevations in BAP and OC, but also non-significant, yet noticeable reductions in urinary DPD during the first three of four months of resistance training in healthy young men (45 minutes, 4 d·wk^-1^, 3 sets of 10 repetitions at 60–80% 1-RM for 7–8 exercises). Ashizawa et al. [67], examining more acute effects of resistance exercise, observed reductions in two resorption markers [urinary DPD and serum tartrate-resistant alkaline phosphatase (TRAP)] within two hours of an intense resistance training workout. Reductions in these markers reached significance (*p *≤ 0.05) on the third and first post-exercise days, respectively. Much more research is needed to investigate the potential for resistance exercise to reduce bone resorption, as well as to determine whether acute shifts in blood and urine chemistry, as in the Ashizawa study, are meaningful in terms of overall bone health.

The present data also did not support the hypothesis that the consumption of 2.4 g protein·kg^-1^·d^-1 ^by women with habitual intakes near the RDA for protein (0.9 g protein·kg^-1^·d^-1^; Table 3) would increase bone resorption or reduce bone formation. As noted previously, one formation marker (serum BAP) did decline with supplementation, but in both groups, while the other (serum OC) showed no change. Regarding bone resorption, it was hypothesized that urinary calcium and DPD would rise in the HP, but not the TC group. Instead, urinary calcium showed no change with supplementation (Figure 5), while DPD levels increased nearly 50% between weeks 8 and 12 in both groups (Figure 6). Ryan et al. [9] reported a similarly unexpected rise in a resorption marker after 16 weeks of resistance training in older men (61 ± 1 y, n = 21), but the use of a different biomarker (serum TRAP) and an older subject sample complicates the ability to draw comparisons.

The lack of a calciuric response by the HP group is perhaps the most puzzling observation, as the protein dose was comparable to that in previous studies that did precipitate such an effect – an effect that has been shown to be consistent, rapid, and sustained [68,69]. It is possible that the phosphorus content of the supplement and the subjects' habitual diet attenuated the hypothesized effects, as phosphorus has known *hypo*calciuric effects [68,70]. A few studies have demonstrated blunted calciuric effects when using meat with a substantial phosphorus content, rather than purified isolates, as the source of supplemental protein [71,72]. The present study used a commercially-available form of purified whey protein, but the supplement did contain a noteworthy amount of phosphorus. An exact value was not available, but an estimate provided by Weider International^® ^indicated that the powder contained about 56 g of phosphorus for every 100 g of protein (personal communication, February 2002). In addition, the subjects' dietary phosphorus intake, exclusive of supplementation (Table 3), was 125% of the RDA for females 19 to 24 years of age [41].

It is also possible that calcium excretion did increase, but through the gut and not the kidney – an increase that would go undetected without measures of endogenous fecal calcium. Previous studies have examined fecal calcium after manipulating dietary protein and have not shown significant changes [68,73], but the majority has measured only total fecal calcium, without distinguishing the endogenous fraction. Heaney et al. [74] have recently generated strong support for possibility that significant fecal losses may have previously gone undetected in studies that did not measure the endogenous fraction. Heaney's group studied 191 adult, female inpatients in a metabolic ward, obtained complete calcium intake, absorption, and excretion data, and demonstrated a significant, inverse relationship between urinary and endogenous fecal calcium. For every 0.41 mg drop in urinary calcium, there was an associated 1.0 mg rise in endogenous fecal calcium. Thus, it may, after all, be critical to measure both urinary and fecal calcium excretion to accurately assess calcium balance.

Still another possibility is that the hypothesized effects on bone turnover may have been offset by the subjects' high calcium intake. Calcium supplementation was considered essential to excluding the effects of calcium deficiency, but the high supplemental dosage, in addition to the subjects' dietary calcium, may have provided enough exogenous alkali material to effectively neutralize any protein-induced acid effects. Shapses et al. [23] have supported this possibility, reporting that an increase in dietary calcium initiated a drop in urinary DPD, and that a large increase in dietary protein had no effect on urinary calcium or DPD when dietary calcium was very high (~1600 mg·d^-1^).

Heaney [75] has simply explained the mechanism through which high calcium intakes may counter the effects of protein excess. Essentially, increased dietary protein stimulates calcium excretion, which in turn stimulates the synthesis and activation of vitamin D to enhance intestinal calcium absorption. If dietary calcium is sufficient, intestinal calcium absorption can be up-regulated and bone resorption is not needed to preserve calcium balance. However, if dietary calcium is inadequate, then an up-regulation of absorption will do nothing to compensate for the increased calcium excretion and bone resorption will ensue.

Finally, it is possible that the relatively uncontrolled timing of the treatment dosages could have influenced the results. While it was recommended that the subjects consume one drink with each daily meal and any additional supplement *ad libitum*, standardization of the timing of supplementation was not possible with these free-living college students and working women. Future studies may want to examine this potential influence.

## Conclusion

The present study provided no evidence that high-intensity resistance training stimulated osteogenic effects, as assessed with serum osteocalcin and bone-specific alkaline phosphatase. It is possible that other biomarkers may have produced different results, and that, given a longer time frame, bone densitometry could detect osteogenic effects. The present study also yielded no evidence that short-term protein supplementation would have osteopenic effects in young adult women. However, the supplementation period was brief and the subjects were healthy, eumenorrheic, calcium-replete women, regularly participating in high-intensity exercise. These characteristics, which may have additive, beneficial effects on bone, are unfortunately not often descriptive of American women, and thus these results must not be taken as justification to perpetuate the common 'more-is-better' mentality toward dietary protein.

## Competing interests

The author(s) declare that they have no competing interests.

## Authors' contributions

NM conceived the study, carried out all described methods, and drafted the manuscript. WS assisted with the design of the study, statistical analyses, interpretation of data, and editing of the manuscript. All authors read and approved the final manuscript.
